# Disruption of specific GDNF receptor subtype signaling impairs cortical neuronal survival in Alzheimer’s brains

**DOI:** 10.1186/1750-1326-7-S1-L26

**Published:** 2012-02-07

**Authors:** Yoshihiro Konishi, Libang Yang, Ping He, Kristina Lindholm, Bai Lu, Rena Li, Yong Shen

**Affiliations:** 1 Haldeman Laboratory of Molecular and Cellular Neurobiology, Sun Health Research Institute, Sun City, AZ 85351, USA; 2 Molecular Endocrinology, Sun Health Research Institute, Sun City, AZ 85351, USA; 3 Section on Synaptic Development, National Institute of Child Health and Development, National Institutes of Health, Bethesda, MD 20892, USA; 4 Research Unit for Alzheimer’s Disease, Department of Clinical Research, National Tottori Medical Center, Tottori 689-0203, Japan; 5 Department of Pediatrics, University of Minnesota, Minneapolis, MN 55445, USA; 6 Center for Hormones Advanced Science and Education, Roskamp Institute, Sarasota, Florida, USA; 7 Center for Advanced Therapeutic Strategies for Brain Disorders, Roskamp Institute, Sarasota, Florida, USA

## Background

Alzheimer’s Disease (AD) Research has long been focusing on Aβ-containing amyloid plaque deposition and tau-containing tangles. However, recent clinical trial outcomes by Aβ-lowering approaches have been disappointing. As an alternative approach, the present study focuses on mechanisms that prevent neuronal loss, another pathological hallmark of AD. Specifically, we have uncovered an unexpected role of glial cell line-derived neurotrophic factor (GDNF) in AD.

## Methods

The location of the Sun Health Research Institute has allowed us access to deceased patients’ brain within 2.5 hours. We have previously developed a method to quickly isolate neurons from postmortem brains, and grow primary neurons in culture for an extended period (Konishi et al., *Am J Pathol.* 2002, 161(5):1567-76). Taking advantage of this unique resource, we have now used primary cortical neurons from rapidly autopsied aged human brains to explore differences between cortical neurons from AD brain and those from non-demented healthy elderly (ND).

## Results

Using this system, we have made a number of interesting findings. First, compared with ND neurons, there is a selective reduction in the expression of specific GDNF family receptor in AD neurons. Second, GDNF and the GDNF family member enhanced the receptor expression in ND neurons but not in AD neurons. Consequently, GDNF supported the survival and promoted the growth of neuronal processes of ND neurons but not AD neurons. Remarkably, introduction of specific receptor to AD neurons restored GDNF enhancement of cell survival. Finally, the survival effects of GDNF- receptor pathway in ND neurons appear to be mediated by the specific signal transduction pathway.

## Conclusion

Taken together, the present study reveals a novel role of GDNF in cortical neuronal survival in normal aged brains. Moreover, our results demonstrate that a key difference between the NC and AD neurons is the presence of specific GDNF receptor, suggesting that up-regulation of GDNF receptor is a potential therapeutic strategy for AD.

